# Non-ionizing radiofrequency electromagnetic waves traversing the head can be used to detect cerebrovascular autoregulation responses

**DOI:** 10.1038/srep21667

**Published:** 2016-02-22

**Authors:** M. Oziel, M. Hjouj, C. A. Gonzalez, J. Lavee, B. Rubinsky

**Affiliations:** 1 Faculty of Life Science, Bar Ilan University, Israel; 2 Medical Imaging Department, Al-Quds University, Abu Dis, Palestine; 3 Instituto Politécnico Nacional-Escuela Superior de Medicina, DF, Mexico; 4 Universidad del Ejército y Fuerza Aérea-EMGS, DF, Mexico; 5 Heart Transplantation Unit, Department of Cardiac Surgery, Leviev Heart Center, Sheba Medical Center, Ramat Gan, Israel; 6 Department of Mechanical Engineering, University of California, Berkeley, CA 94720 USA

## Abstract

Monitoring changes in non-ionizing radiofrequency electromagnetic waves as they traverse the brain can detect the effects of stimuli employed in cerebrovascular autoregulation (CVA) tests on the brain, without contact and in real time. CVA is a physiological phenomenon of importance to health, used for diagnosis of a number of diseases of the brain with a vascular component. The technology described here is being developed for use in diagnosis of injuries and diseases of the brain in rural and economically underdeveloped parts of the world. A group of nine subjects participated in this pilot clinical evaluation of the technology. Substantial research remains to be done on correlating the measurements with physiology and anatomy.

Medical imaging for diagnosis is not available to a large segment of the world population. This medical problem cannot be ameliorated through well intentioned donations of state of the art medical equipment, because these devices required trained physicians, who are not available to service that segment of the population. This study is part of our decade long international collaboration effort aimed at developing affordable medical diagnosis technologies to reduce medical care cost in remote and impoverished parts of the world^1^,^2^,^3^,^4^,^5^,^6^,^7^,^1^,^2^,^3^,^4^,^5^,^6^,^7^. Our goal is to develop medical technologies that, while using advanced scientific concepts, are inexpensive, robust and do not require advanced medical training for use.

In this study we introduce a new technology that is inexpensive and does not require advanced medical training, for possible use in the diagnosis of cerebrovascular diseases. The physiological basis of the technology is the phenomenon of cerebrovascular autoregulation (CVA). The CVA concept is a simplified term that describes the complex response of the cerebrovascular blood flow (CBF) to the interplay between the physiological and functional needs of the brain metabolism and those of the rest of the body^8^,^9^,^10^,^11^,^12^. A number of mechanisms have evolved to safeguard the brain against dangerous fluctuations in blood supply and optimize the response to metabolic requirements of the brain. They include: metabolic regulation, myogenic regulation and, neurogenic regulation^13^. The overall effect is the CVA. Many excellent reviews of this field exist, e.g.^8^,^12^,^13^,^14^,^15^,^16^,^17^,^18^. Evaluation of CVA is a valuable tool for diagnosis of the normal function of the brain and of various diseases related to the brain and to blood flow^15^,^19^,^20^.

Cerebral autoregulation can be characterized in a static and dynamic context, by assessing static cerebrovascular autoregulation (sCVA) and dynamic cerebrovascular autoregulation (dCVA). While our technology can be applied to both sCVA and dCVA based diagnosis, the focus of this paper is the dCVA. The concept of dynamic CVA, is based on the observation that when the brain normal homeostasis is disturbed by a sudden change, the CBF adjusts rapidly, usually within 2–10 seconds, to a new steady state value^13^,^21^. The principle of dCVA measurement is based on evaluation of the stimulus-response process that aims to maintain adequate and stable cerebral blood flow^13^,^22^,^23^,^24^. Various stimuli are used in dCVA. Methods involving external stimuli include deflation of large cuffs placed around tights^21^ and isometric exercises^14^. Stimuli such as changes in posture, head tilt angle, or between standing and sitting are simple to implement and well tolerated by the elderly^14^,^25^,^26^. Changes in the blood level of CO_2_, such as through breath holding or occlusion of the carotid artery are also commonly used as stimuli^27^. Several different techniques are used to measure the physiological responses to CVA stimuli, including PET, MRI, Xenon computed tomography, and Transcranial Doppler ultrasonography (TCD)^16^. An excellent review of different techniques to measure the physiological responses to the CVA stimuli can be found in^16^. It is evident that while the application of the stimuli is simple and does not require advanced medical training, monitoring the response requires expensive devices and advanced radiological skills and training: all of which are often not readily available in rural and impoverished parts of the world. This study introduces a new technology aimed to measure responses to CVA stimuli, that may be more suitable for use in resource limited parts of the world. The technology is based on non-contact electromagnetic measurements across the brain.

It is well established that the complex electrical impedance of tissue, can be used for tissue characterization^28^,^29^,^30^. This property of tissues is used in a tissue imaging modality known as electrical impedance tomography (EIT)^31^,^32^. EIT uses an array of electrodes to inject subsensory currents and measure the resultant voltages. The data are used to reconstruct a map of the electrical impedance of tissue that forms the image. Bioelectrical measurements by electromagnetic induction with non-contact electrical coils are considered as a valuable alternative to contact electrode measurement^33^,^34^,^35^. Inductive measurement does not require galvanic coupling between the electrode and the skin or the tissue under measurement. In the case of the brain, the skull does not represent a barrier for the magnetic field. Non-contact measurements have also found application in developing an alternative technique for electrical imaging of tissue magnetic induction tomography (MIT) and its different variants^34^,^36^. The various dCVA stimuli yield physiological changes in the brain that are characterized by changes in overall brain tissue composition and structure; in particular changes in the relative volume of blood, cerebrospinal fluid and intracellular and extracellular fluid. These changes affect the electrical properties of tissue and may be visible with EIT or MIT imaging. However, medical imaging, which provides detailed information on tissue structure, is not appropriate for use in economically disadvantaged parts of the world, because it is expensive and requires experts.

Our design goal is to develop a device that is simple to use, robust, inexpensive, could be conveniently placed in parts of the world lacking medical infrastructure and which can be used to evaluate the brain response to dCVA stimuli. The hypothesis behind this study is that non-contact measurements of the bulk electromagnetic properties of the entire brain, or of large parts of the brain, are sensitive enough to detect a response to a dCVA stimuli. If this hypothesis is correct, than our diagnosis goals of evaluating the dynamics of CVA could be achieved with a device that measures with non-contact electromagnetic induction the electromagnetic properties of the entire brain. To examine this hypothesis we conceived of a simple, inexpensive and easy to use device that consists of two electromagnetic coils placed on the skull in such a way that the brain is braced by the coils. One coil serves as the emitter and the second as the receiver. As the electromagnetic waves traverse the brain, they undergo a phase shift and changes in amplitude. Comparing the electromagnetic waves in the emitter and receiver are a measure of the bulk electrical impedance of the brain. Changes in the receiver electromagnetic waves should reflect bulk changes in the brain electrical impedance in response to a CVA stimulus. Therefore, the primary goal of this small scale pilot study is to determine if this device and measurement technology is sensitive enough to detect a response to a typical CVA stimuli, and if the measurements are consistent and repeatable. A small group of nine subjects participated in this pilot clinical study. The goal of the study is solely technological. No attempt was made to generate a physiological understanding of the significance of the measurements. A study on the physiological significance of our measurements requires substantially more research and will be done in the future.

## Results

**Figure 1:**
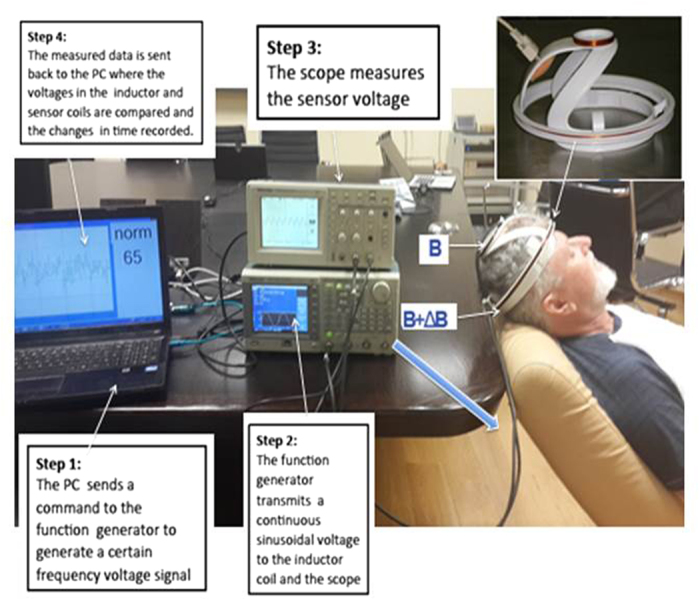
The non-ionizing radiofrequency electromagnetic waves experimental system. Top right corner insert, the head coil device. The experimental system consists of a circuit made of a function generator, connected to the head coil, and to the oscilloscope, both connected to a laptop. The figure shows the elements of the system and the head coil on an examinee. (Photo used with BR permission).

Figure 1 is a photograph of the experimental system used in this study. The system consists of two head coils, on the head of a subject, a signal generator and an oscilloscope. The two head coils, an emitter and a receiver are placed near the skull in such a way that they brace the brain of the subject. The measurements consist of monitoring the amplitude change and the phase change between the electromagnetic waves in the emitter and receiver to evaluate changes in the bulk electromagnetic properties of the brain tissue, during and after the delivery of a CVA stimulus. A detailed description of the system is given in the materials and methods section.

**Figure 2:**
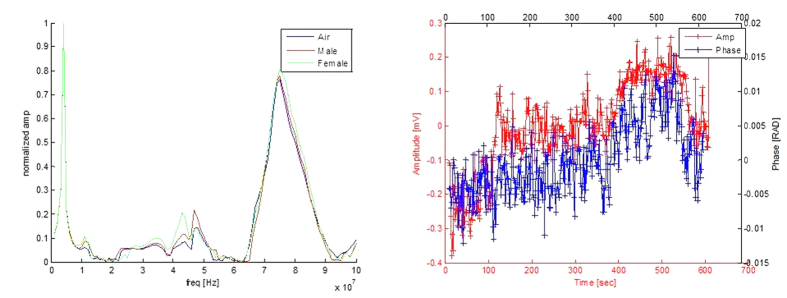
Characterization of the experimental system. Left panel shows transfer functions across the coil as a function of frequency. The panel displays three curves, from two volunteers wearing the coils and a measurement in air. The resonance frequencies are evident. (The max amplitude of the 3 measurements was normalized to 1). Right panel shows the system measurement across the coils in air as a function of time.

Figure 2 shows results from the system calibration tests. The left panel of Fig. 2 shows the frequency dependent response of the head coils, in air and across the heads of two volunteers, one female and one male. It was obtained by connecting the top (induction) coil to the function generator and the oscilloscope to the bottom (sensor) coil. The input signal was a 5 V peak-to-peak sinusoidal signal at various frequencies. The panel shows the amplitude of the output voltage relative to the input voltage as a function of the signal frequency. The panel shows that the highest resonance frequency of our system is around 78.1 MHz. Consequently, in all the experiments the input signal delivered by the function generator was a 5 V peak-to-peak sinusoidal signal at a frequency of 76 MHz. The right panel of Fig. 2 shows the amplitude (V) and phase angle (radians) differences between the inductor and sensor coil measurements, for the 76 MHz input signal, in air, as a function of time. The left scale is the amplitude change and the right scale shows the phase shift. The figure shows that, in air, the voltage fluctuates in a range of +/−0.25 mV and the phase fluctuation is in the range of +/−0.015 radians. This is the device resolution.

**Figure 3:**
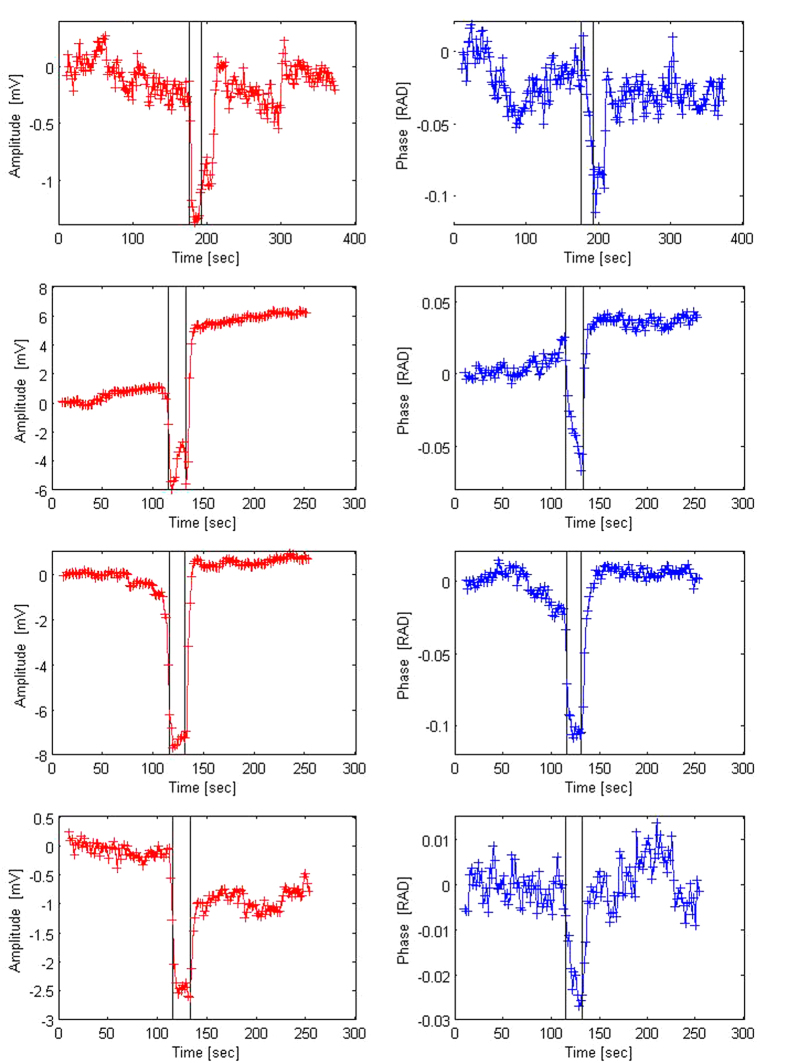
Change in amplitude (V) and phase (RAD) across the head as a function of time, during the left carotid artery compression test. The results are from four different volunteers. The dark stripes indicate the times at which the compression was delivered. From top to bottom, subject (**A–D**).

Figure 3 shows results from experiments with a CVA stimulus in which the left carotid artery was compressed for 15 seconds, as described in the materials and methods section. The subjects were placed with the torso at 30 degrees to horizontal (see Fig. 1) and were at rest for two to three minutes before and after the carotid artery compression. The compression was delivered by a trained physician. The figures show the changes in amplitude (V) (left columns) and the phase shift (radians) (right columns) between the emitter and the receiver as the electromagnetic waves traverse the head, during the carotid artery compression. The grey shaded strip in the figures represent the approximate times in which the carotid artery was compressed. The reason for the designation “approximate” is described in the materials and methods section. The results are typical to all the subjects and repeats. Each one of the four pairs of panels, are from a different subject.

Several observations emerge from Fig. 3. The change in amplitude has a qualitatively similar pattern in all the experiments performed in this study. The pattern consists of a sharp decrease in amplitude with the start of the compression. The change is maintained throughout the application of the compression. When the compression is stopped, there is a slower, almost exponential increase towards the original value. The phase shift has also a qualitatively similar pattern in all the experiments. A significant change occurs in the phase shift with the application of the compression followed by a slower return towards the normal values with the release of the compression.

**Figure 4:**
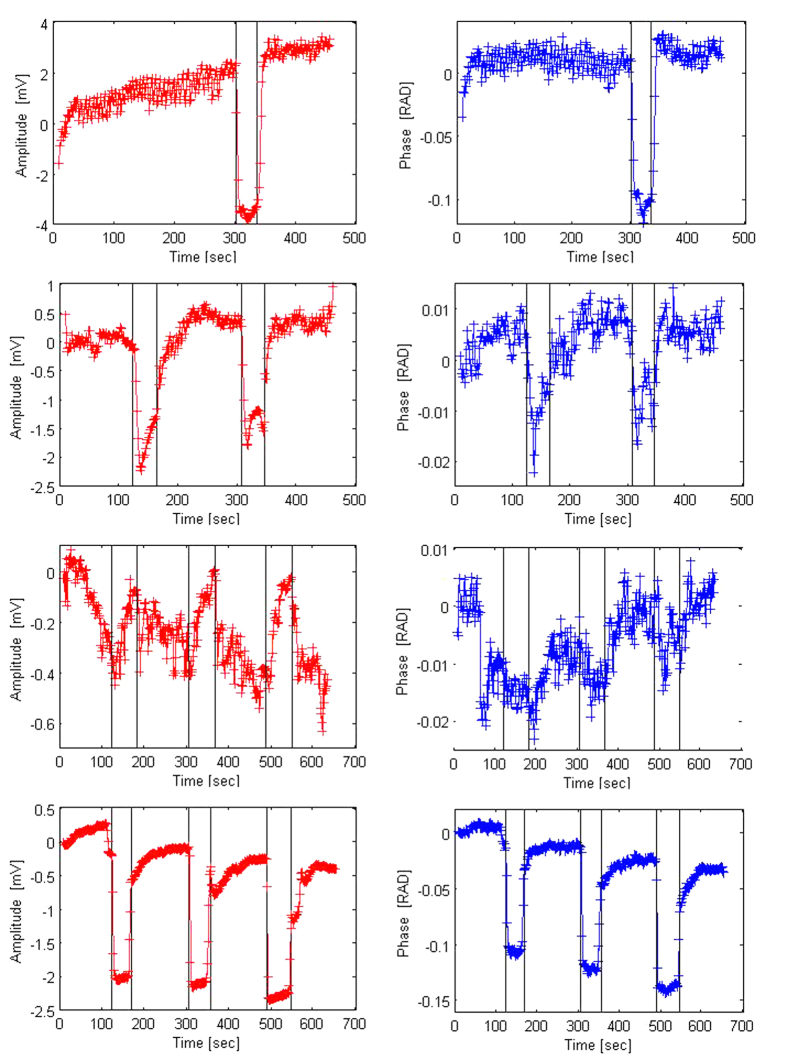
Change in amplitude (V) and phase (RAD) across the head as a function of time, during a BHPV test. Top three rows are for the same subjects. Top row panels show results for a volunteer on a flat bed with the torso and the head at 30 degrees to the rest of the body. Second from top panels show results for the same volunteer sitting on a chair with the torso and head vertical to the ground (90 degrees). Third from top panels show results for the same volunteer in a supine position. Bottom panel shows results from a different subject on a flat bed with the torso and head at 30 degrees to the rest of the body. The dark stripes indicate the approximate times at which the test was performed.

Figures 4 show the change in amplitude (V) and phase (radians) between the emitter and receiver coils, as a function of time, during tests with a CVA stimulus involving a Breath Holding with Partial Valsalva (BHPV) procedure. The details of the BHPV stimulus are described in the materials and methods section. The gray vertical bands represent the approximate times in which the BHPV stimulus was delivered, as described in the materials and methods section. The left columns show the changes in amplitude and the right columns show the changes in phase shift, during the delivery of the BHPV stimuli. The figures compare BHPV tests done under different conditions.

The first three rows of panels from the top of Fig. 4 are for one subject under three different test conditions. The first row is for the subject with the torso at an inclined angle of 30 degrees, as shown in Fig. 1. Only one BHPV stimuli was administered in the test whose results are depicted in the top row. The observed response is typical to all the experiments. Both the amplitude and the phase shift change noticeable with the delivery of the BHPV stimulus and remain at the new level throughout the delivery of the stimulus. Both the amplitude and the phase return towards the initial values after the stimulus stopped. The second row from the top shows results of an experiment with a subject sitting at 90 degrees. Here, the subject delivered two BHPV stimuli, one after the other, separated by a three minutes rest. Two observations emerge from the panels in this row. First, the response to the two consecutive BHPV stimuli, in the same subject, is qualitatively and quantitatively similar. Both the amplitude and the phase shift change noticeable with the delivery of the BHPV stimulus and remain at the new level throughout the delivery of the stimulus. Both the amplitude and the phase return towards the initial values at the end of the stimulus. Second, the measurements have larger fluctuations around the local mean during the rest period before, between and after the BPHV stimuli, than in the experiments with the subject torso at a 30 degrees angle. This is a pattern that we have observed in all our experiments. The third panel from the top shows measurements on a subject laying horizontally. Here the subject delivered three BHPV stimuli one after the other, each stimulus separated from the other by a rest period of two minutes. While the results suggest a pattern, the fluctuations around the mean prior to the experiments and during the rest period are too large to conclude that the effect of the BHPV stimulus is measurable. This effect of position on measurement was consistently observed in all the experiments. The bottom panel shows result from a different subject than the one in the top three panels. The subject was on a flat bed with the torso at 30 degrees to the horizontal; i.e. the same condition as the measurement taken from the subject whose data is displayed in the top row. This subject self-administered three BHPV stimuli, one after the other, each separated from the other by a two minutes rest period. The response to the BHPV stimulus in the subject whose data is displayed in the top row is similar to the response to the stimulus in the subject whose data is displayed in the bottom row. The response to the three different BHBP stimuli in the subject whose results are displayed in the bottom row is the same for all the stimuli.

A consistent data pattern emerges from all the measurement plots in Fig. 4. Similar to the response to the carotid artery compression, we find that the change in amplitude has a qualitatively similar pattern in all the BHPV stimulus tests. The pattern consists of a sharp, decrease in amplitude with the start of the BHPV stimulus. The change is maintained throughout the duration of the BHPV procedure. When the stimulus stops, there is a slower, almost exponential, increase towards the original value. The phase shift has also a qualitatively similar pattern across all the BHPV experiments. A significant change occurs in the phase shift with the application of the BHPV stimulus, followed by a return towards the original value when the stimulus ends.

## Discussion

The principle of CVA based diagnosis is based on evaluation of the stimulus-response process that aims to maintain adequate and stable cerebral blood flow^13^,^22^,^23^,^24^. The primary goal of this study is to evaluate the feasibility of monitoring responses to typical CVA stimuli using a technology that transmits non-contact non-ionizing radiofrequency (NIRF) electromagnetic waves, across the brain. The goal of this pilot study is limited to an examination of the measured CVA response sensitivity and repeatability.

The introduction provides references to various CVA triggering stimuli. Since our primarily goal is to develop a technology that is simple enough to be administered by examiners with limited medical training in economically disadvantaged parts of the world, we have chosen for our first tests two, simple to administer, well established stimuli^27^. One stimulus is the temporary occlusion of one carotid artery. A second stimulus consists of breath holding through a deep aspiration ending with a small forced Valsalva like exhalation. Training for compression of one carotid artery is simple and can be self-administered. The breath holding with partial Valsalva (BHPV) or modified Valsalva technique, is well tolerated by CVA studies subjects^37^. BHPV is commonly used in conventional CVA research and can be easily self-administered. It is naturally occurring in many every day activities such as heavy weight lifting and toilet use^8^.

Figure 3 shows results from experiments with a CVA stimulus in which the left carotid artery was compressed for 15 seconds. The pattern, described in the results section, is qualitatively similar among all the subjects and quantitatively similar in repeats for the same subject. The changes in amplitude and the phase shift due to the compression are an order of magnitude larger than the system resolution and noise.

Figure 3 illustrates several aspects of the technology, that were the focus of this pilot study. They demonstrate that the amplitude change and phase shift change measurements are sensitive enough to detect changes due to a carotid artery compression stimulus. The data also shows that when the stimulus ends the CVA is activated and starts returning the fluid distribution in the brain to its original value. The biophysical principle of this technology, as discussed in greater detail in the biophysical principle section in the materials and methods section, is based on the fact that the complex impedance of blood is different from the complex impedance of brain tissue and from the complex impedance of plasma serum. Therefore, when a change in the relative volume of blood, brain tissue and cerebrospinal fluid occurs, the bulk complex impedance of the brain changes. Since the change in amplitude and phase of the electromagnetic waves traversing the brain are a function of the bulk complex impedance of the brain, any change in the complex impedance of the brain produces a change in the amplitude and phase of the waves across the brain. While these facts are well established, the main motivation for this study was to determine if our technology is sensitive enough to detect changes due to a carotid compression stimulus. Repeatable results from nine subjects, demonstrate that the technology is sensitive enough to measure changes in the blood/spinal fluid/brain tissue composition due to carotid compression stimuli and that the changes are repeatable and similar between subjects.

Figure 4 shows that the application of the BHPV stimulus causes a decrease in amplitude and an increase in phase shift, throughout the administration of the stimulus. An important observation is that the response is affected by the position of the subject. The best quality results are for a subject with the torso at 30 degrees to horizontal, followed by that of subjects in a sitting position with the head and back at 90 degrees to horizontal. The results for the supine laying volunteer are of low quality with the fluctuations around the mean during the resting period and before the test on the order of magnitude of the response to the BHPV stimulus. This is consistent with the findings of other researchers who show that the CVA response to the Valsalva maneuver is affected by posture, with the back at 30 degrees producing a better response than the supine posture^38^. This is most probably secondary to the highest mean central venous pressure in the supine position, which causes the diminishing of the trans-cranial vascular pressure gradient (mean arterial pressure minus mean central venous pressure) and hence the diminished CVA. The consistent effects of position on the measurements, is another indication that they are affected by physiology. The response to BHPV at a 30 degrees to horizontal posture is clear and unambiguous.

When the BHPV test ends, the cerebrovascular condition returns rapidly, within a few seconds to the original state. This fast temporal response to a BHPV stimulus is also consistent with other reports in the literature^13^,^21^.

The primary physiological effect of breath holding, is hypercapnia, the increased levels of CO_2_ in blood and the consequent expansion of blood vessels in the brain and the increased blood flow to the brain^39^. The increase in the diameter of blood vessels is the equivalent of an increase in the extracellular blood volume. As indicated in the discussion on the carotid compression stimulus, the change in the ratio between blood, cerebrospinal fluid and brain tissue yields a change in the relative reactive component of the bulk brain tissue electric impedance. By measuring the change in amplitude and phase of electromagnetic waves traversing the head, our technology measures the changes in bulk brain impedance and the ratio between blood volume, spinal fluid and brain tissue volume^5^. The goal of this study was to determine if the changes in blood volume and distribution during a BHPV stimulus can be detected with our technology. The key conclusion from this part of the study is that our technology is sensitive enough to detect changes in the brain due to a BHPV stimulus. Figure 4 top and bottom rows demonstrate the sensitivity and the repeatability of these measurements both between different subjects and for repeated measurements of the same subject.

In summary, Figs 3 and 4, show that the amplitude and phase shift measurements are sensitive enough to detect changes in the bulk brain composition due to both carotid artery compression and BHPV stimuli. The data also shows that when the stimulus ends the CVA is activated and starts returning the fluid distribution in the brain to its original value. We believe that analyzing the dynamics of the return to the original value will be an important element in the CVA diagnosis. In general, correlating the radiofrequency electromagnetic wave measurements with physiological data in the brain, holds promise in understanding CVA and will facilitate its use in brain diagnosis.

## Conclusion

The goal of this study was to introduce a new technology for measurement of the brain response to typical stimuli used in CVA evaluation. The technology, which measures low energy non-ionizing radiofrequency electromagnetic waves transmission through the brain can detect relative changes in blood volume, cerebrospinal fluid volume and brain tissue volume through their effect on the complex electrical impedance of the brain. This study is focused on technology evaluation. We tried to determine if the technology is sensitive enough to detect CVA type stimuli induced changes in the brain and to verify the repeatability of these measurements. The study demonstrates that this simple and inexpensive technology is sensitive enough to detect changes due to typical CVA examination stimuli and the results are repeatable qualitatively and quantitatively.

This is a very limited clinical study with a small number of subjects. No attempt was made to correlate the readings with physiology and the anatomy. Obviously a further study that correlates NIRF measurements with physiology and anatomy is required. While this pilot study is limited, the results suggest that this technology has value in many applications. Extrapolating widely, there is a possibility that measuring the complex impedance in parts of the brain could produce information comparable to that produced by PET or other complex imaging devices at a small fraction of the cost. This would be consistent with our goal of developing a diagnostic technology of the brain for use in remote and poor parts of the world and reducing the cost of medicine.

## Materials and Methods

### The biophysical principle

The complex impedance of biological tissue displays changes with tissue type, composition and with frequency. The changes with frequency occur in the range of frequency from DC to GHz, in three distinctive dispersions^40^. The electrical permittivity and conductivity of the three main dielectric dispersions have been labeled α, β, γ. They occur at increasing frequencies from DC trough MHz to GHz, respectively. The α-dispersion is caused by the relaxation in the counter-ion atmosphere surrounding the charged cell membrane surface, the β-dispersion is produced by Maxwell–Wagner relaxation, an interfacial relaxation process occurring in materials containing boundaries between two different dielectrics and the γ-dispersion by the relaxation of free water within tissues. The use of bioelectrical impedance measurements for tissue characterization was suggested already half a century ago (as reviewed in^41^). Bioelectrical measurements by electro-magnetic induction with non-contact electrical coils are a valuable alternative to contact electrode measurement^33^. Inductive measurement does not require galvanic coupling between the electrode and the skin or the tissue under measurement. In the particular case of brain electromagnetic properties measurement, the skull does not represent a barrier for the magnetic field^33^. This is why we have chosen non-contact electromagnetic measurements for our technology. As indicated in the introduction, the electrical properties of tissue are used to produce an image of the tissue in the technologies known as Electrical Impedance Tomography and Magnetic Induction Tomography. The novelty of our work is that instead of producing an image from transcranial electromagnetic measurements, we seek to produce medical diagnostics from measurements that depend on the changes in electromagnetic properties of tissue (brain) in a composite volume of tissue (the entire brain or certain volumes in the brain). This leads to a very simple, inexpensive and robust device, made of an emitter coil and a receiver coil placed across the head to brace the brain. The measurements entail the changes in amplitude and phase between the sinusoidal signals in the emitter and receiver. The significance of the data gathered in this experiment is best understood through Table 1. Our measurements reflect the changing electromagnetic properties of a volumetric composite of various tissues. The measurement will obviously depend on the properties of each component and their relative volume in the composite. Table 1 shows that at frequencies between 25 MHz and 100 MHz, the electrical conductivity of brain tissue is substantially different from that of human serum or blood and that close to 100 MHZ the properties of serum are substantially different from those of blood. Therefore, from extrapolation, we anticipate that measurements at about 80 MHz would capture changes in bulk properties of the brain caused by relative changes in blood volume, cerebrospinal fluid and brain tissue. It should be emphasized that multifrequency measurements may provide additional insight. However, at this stage, our goal was to determine if the technology is sensitive enough to detect any changes in the brain caused by a CVA stimulus. In summary, the basic biophysical concept underlining our technology is that, since the electric properties of brain tissue, blood and cerebrospinal fluid are different, the bulk electric properties of the brain will reflect the relative volume of these different types of biological material. Changes in the relative volume of these types of tissue in response to a CVA stimulus, will express themselves as changes in the bulk electromagnetic properties of the brain. This in turn, will affect the electromagnetic waves traversing the brain between an emitter and a receiver across the brain providing, thereby, a means to detect the bulk response of the brain to a CVA stimulus. While the biophysical principle is obvious, the key question that this work seeks to determine is if the measurements are sensitive enough to detect these changes.

A schematic of the human head/coil geometrical configuration used in this study is shown in the insert in Fig. 1. The device is very simple. It consists of two coils of different radii in an inductor-sensor arrangement. The coils are coaxially centered. The brain (head) is placed between the coils. An alternating current, *e*^*jwt*^ is injected into the inductor coil. The current generates a primary magnetic field B that is detected by the sensor coil. The volume of tissue confined between the coils produces a perturbation of the primary magnetic field, (Δ***B***) The perturbation is a function of the complex impedance of the brain tissue in the volume between the coils. The perturbation is evaluated by comparing the magnetic field in the sensor coil (***B *****+ **Δ***B***) , to the primary magnetic field, B. Changes in the magnetic field represent volumetric changes in the brain composition complex impedance. A robust way to detect changes in the magnetic field is to measure the voltage phase shift and the amplitude change between the inductor coil and the sensor coil. A simple way to measure the phase shift and amplitude change is through a “voltage relative to voltage” arrangement^42^. While the device used here was designed to acquire data from measurements through the entire volume of the brain^5^, similar technologies could be used for parts and specific volumes of the brain^43^. Furthermore, in this first study we have used only one frequency. However, since the electrical properties of the tissue change with frequency, multifrequency measurements could produce additional insight.

### CVA monitoring system

The CVA monitoring experimental system is shown in Fig. 1. It consists of four modules: head-coil unit, function generator, oscilloscope and a laptop, that functioned as a microprocessor. The head-coil unit consists of two concentric coils: an inductor coil with radii of R1 = 3.2 cm (top) and a sensor coil with radii of R2 = 11 cm (bottom), separated by a distance of 10 cm. Both coils were made from ten turns of magnet wire AWG22 rolled on an ergonomic plastic harness specifically designed for an adult human head (Fig. 1 photographic insert, top left corner). The coil inductances, calculated from Faraday’s law, are approximately 67.4 and 796.4 μH for the inductor and sensor coils, respectively. The estimated mutual inductance coefficient is approximately M = 72.8 μH. The function generator is a 1 GS/s, Tektronix 3102 dual channel unit. The function generator is connected to the head coils with coaxial cables and BNC connectors and to the laptop with a USB cable. The sensor coil is connected to a 1 GS/s digital real-time oscilloscope Tektronix TDS 210 (2 channels). The oscilloscope connects to a laptop via RSS232 to a USB cable. As any RF measurement contains noise, a very effective tool to reduce the noise is averaging the signal over time. To obtain the best and most accurate signal, we can, our system takes for each data point 128 measurements and averages the data. This is the maximum averaging value that our scope provides. The function generator triggers the scope via a direct connection to the second scope channel. The laptop controls both the function generator and the oscilloscope and samples the measured data from the oscilloscope. For each sampling, the laptop collects 2000 data points of the measured signal and calculates the amplitude change and the phase shift. Our current experimental system’s time resolution is about 1 sample per second, as a result of its inherent slow speed of communication between the oscilloscope and the computer. This slow speed will be significantly increased in our following hardware upgrades. A higher sampling speed is required to correlate the kinetics of CVA response to physiological events. The following observation may be useful for researchers attempting to repeat our study. In the original display of data, we found that the amplitude change sign was consistent in all measurements, while the phase measurements sign were not consistent; at times they were positive and at times negative. A further in depth analysis has shown that the variations in phase shift signs were a technical artifact. Because the frequencies of both signals from the emitter and receiver are the same, the scope was sometimes triggered from the upward part of the curve and in other times from the downward part of the curve, resulting in the appearance of different angles. The displayed phase shift figures show the absolute change only.

It should be emphasized that the experimental system in Fig. 1, was assembled from individual, generic, standalone devices. However, a final version of our device should consist of a simple electronics box with an on/off button, that could be part of a standalone, battery operated, head-coil assembly. The measurements focus on the changes in amplitude and the phase shift. We have not used a common baseline in all the measurements, and did not try to normalize the data to the subjects physiological dimensions, as done by us in^5^. Therefore the data to notice is the actual values for change in voltage, in units of mV , and the actual values of the phase shift in units of radians.

### Experimental Design

Ethics Statement - The study was conducted according to the principles expressed in the Declaration of Helsinki, and was approved by the “Research Ethics Committee” of Al-Quds University. Volunteers were informed in the presence of witnesses about the experimental protocol as well as its potential risks. The inclusion criteria were: female and males in excellent health. Nine subjects, five males and four females with an age range from 28 years of age to 68 years of age participated in this study, with informed consent.

The goal of this study is to determine: a) if the CVA monitoring technology introduced here is sensitive enough to detect cerebrovascular autoregulation induced changes in the brain, b) if these changes are consistent and repeatable between measurements and among subjects. Our goal is to facilitate the use of our technology in places that for geo-economical reasons lack advanced medical facilities. To this end we chose stimuli that could be self-administered and that do not require advanced technology. We employed the following stimuli in our study:

- Carotid artery occlusion^44^. This test was delivered to a subject placed at 30 degrees from the horizontal (see Fig. 1). Following two minutes of baseline CVA recording, the right carotid artery of the examinee was manually compressed by an expert physician for 15 seconds, followed by continued CVA recording for another two minutes.
- Breath holding with partial Valsalva (BHPV). In these experiments, the examinee was placed into one of the following three positions: a) supine, b) supine with the back and head at 30 degrees from horizontal and c) upright sitting with the back and head at 90 degree from horizontal. At that point the volunteer was asked to maintain normal breathing for a few minutes. This was followed by a request to take a deep aspiration ending with a small forced Valsalva like exhalation. The volunteer maintained this condition for a predetermined period of time, after which he resumed normal breath. This procedure is known in the literature as breath holding with partial Valsalva (BHPV) or a modified Valsalva technique, and is well tolerated by the volunteers^37^. BHPV is commonly used in conventional cerebrovascular reactivity research and can be easily self-administered. In fact, it is naturally occurring in many daily activities such as heavy weight lifting and toilet use^8^. The BHPV tests were performed in several ways: as a single stimulus or as two or three BHPV stimuli separated by a rest period.

The computer data recording and the supervision over the CVA stimuli delivery were done by different individuals. The individual in charge of data recording instructed the individual in charge of the stimulus when to start and end the test and marked the time on the computer. These markers form the boundaries of the gray area in the results display. However, while the markers represent the input from the individual in charge of data recording, the data display represent the actions of the individual in charge of delivering the stimulus. The discrepancy is due to the differences in time response between these two individuals.

The volunteers were requested to avoid any movements, throughout the test. However, there may be some head movements during the test. We will distinguish between two types of head movements: A slight movement, defined as a head movement inside the coil of less than 3 mm, and a large head movement, defined as a movement of the coil and the head together of more than 3 mm. While slight head movements result in amplitude fluctuations of about 1 to 3 mV, large head movements generate much larger signal fluctuations, of more than 15 mV. In our analysis, we have ignored the slight head movements but deleted the large head movement.

The volunteers reported no discomfort during or after the experiments.

## Additional Information

**How to cite this article**: Oziel, M. *et al.* Non-ionizing radiofrequency electromagnetic waves traversing the head can be used to detect cerebrovascular autoregulation responses. *Sci. Rep.* **6**, 21667; doi: 10.1038/srep21667 (2016).

## Figures and Tables

**Table 1:** Electrical conductivity (S/m) at specific frequencies for brain tissue, human serum and blood^30^,^50^.

Frequency Tissues/fluid	25 MHz	100 MHz
Brain (grey matter)	0.4	1
Human Serum	1.03	1.14
Blood	1.09	1.3
