# Corrigendum: Non-ionizing radiofrequency electromagnetic waves traversing the head can be used to detect cerebrovascular autoregulation responses

**DOI:** 10.1038/srep23875

**Published:** 2016-04-20

**Authors:** M. Oziel, M. Hjouj, C. A. Gonzalez, J. Lavee, B. Rubinsky

Scientific Reports 6: Article number: 2166710.1038/srep21667; published online: 02222016; updated: 04202016.

In this Article, J. Lavee is incorrectly listed as being affiliated with ‘Faculty of Life Science, Bar Ilan University, Israel’. The correct affiliation is listed below:

Sackler Faculty of Medicine, Tel Aviv University, Tel Aviv, Israel.

